# The Standing Forward Flexion Test in Manual Therapy: A Critical Review and a Functional Reinterpretation

**DOI:** 10.7759/cureus.101225

**Published:** 2026-01-10

**Authors:** Saverio Colonna, Fabio Casacci

**Affiliations:** 1 Rehabilitation Medicine, Spine Center, Bologna, ITA; 2 Research and Development, Osteopathic Spine Center Education, Bologna, ITA

**Keywords:** clinical interpretation, functional assessment, lumbopelvic rhythm, manual therapy test, myofascial dysfunction, palpatory reliability, pelvic asymmetry, pelvic palpation, sacroiliac joint, standing forward flexion

## Abstract

The standing forward flexion test (SFT) is widely used in manual and osteopathic practice as a palpatory assessment traditionally interpreted as a test for sacroiliac joint dysfunction. Despite its popularity, the clinical meaning of SFT findings remains controversial, particularly in light of evidence demonstrating the minimal intrinsic mobility of the sacroiliac joint, high prevalence of pelvic morphological asymmetries, and multifactorial determinants of posterior superior iliac spine (PSIS) motion. Several studies have questioned the clinical utility of the SFT, reporting poor inter-examiner reliability when the test is performed without standardized procedures or adequate examiner training. This critical narrative review aims to re-examine the SFT by clearly distinguishing issues of reproducibility from those of interpretative validity, and by integrating biomechanical, anatomical, and myofascial evidence. The available literature indicates that, following specific and standardized training, the SFT may achieve acceptable levels of reproducibility and inter-examiner agreement. However, PSIS asymmetries observed during trunk flexion appear to reflect global adaptations of the lumbopelvic complex, including pelvic morphology, hip mobility, and cranial and caudal myofascial tension, rather than isolated sacroiliac joint motion. From this perspective, the SFT shows important limitations in terms of content, construct, and criterion validity when used as a diagnostic test for sacroiliac pathology. Instead, it may be more appropriately interpreted as a functional assessment of lumbopelvic rhythm during the transition from trunk extension to flexion. Abandoning a binary positive/negative interpretation in favor of a graded, context-dependent evaluation may enhance the clinical relevance of the SFT when integrated into a comprehensive biomechanical assessment.

## Introduction and background

The pelvis represents a key junction within the human body; accordingly, the literature describes numerous palpatory landmark acquisition techniques aimed at identifying its spatial position and physiological or non-physiological movement. The standing forward flexion test (SFT), also referred to as the Vorlauf test (of untraceable origin) and the Piedallu test [1], is one of the most commonly adopted assessments across various manual medicine disciplines.

The SFT is a commonly used palpatory test in manual therapy and osteopathy, traditionally employed to assess asymmetries in lumbopelvic motion during trunk flexion. During the test, the examiner palpates the posterior superior iliac spines (PSIS) and observes their relative movement as the subject bends forward. A test is traditionally considered “positive” when one PSIS appears to move cranially and anteriorly before or more than the contralateral side. Despite its apparent simplicity and widespread clinical use, the SFT has long been characterized by a fundamental paradox: it is routinely taught and applied in clinical practice, yet repeatedly reported as poorly reliable in the scientific literature. This discrepancy reflects the fact that the SFT has been widely adopted in osteopathic and manual therapy practice primarily on historical and educational grounds, rather than based on robust empirical validation.

Early osteopathic literature proposed interpretative models linking asymmetric PSIS motion during trunk flexion to sacroiliac joint dysfunction; however, these models were largely conceptual and descriptive in nature. Over time, such interpretations became embedded in clinical teaching and practice, often preceding systematic evaluation of their reliability, validity, and biomechanical plausibility.

For this reason, the present review distinguishes between the historical rationale underlying the clinical use of the SFT and the current evidence regarding its reliability, validity, and biomechanical interpretation, critically examining these dimensions as interrelated but conceptually distinct. The present review deliberately focuses only on the SFT. This choice is not intended to imply superiority over other pelvic assessment procedures, but reflects the historical and educational relevance of the SFT as the first pelvic motion test described in osteopathic literature and the one most consistently taught in osteopathic and manual therapy training programs. For this reason, the SFT represents a paradigmatic example through which broader issues of palpatory reliability, test interpretation, and clinical meaning can be critically examined. Accordingly, this review does not aim to provide a comprehensive comparison of all pelvic motion tests, nor to systematically address alternative landmarks such as the anterior superior iliac spine or assessments performed in seated or prone positions. These procedures raise additional biomechanical and methodological considerations that fall outside the specific scope of the present analysis and would require a separate dedicated review. The focus on the PSIS and on the standing execution of the test reflects their central role in the traditional performance and interpretation of the SFT as commonly applied in clinical practice. This review adopts a critical narrative approach rather than a systematic methodology. Given the heterogeneity of study designs, outcome measures, and interpretative frameworks within the available literature, a systematic review or quantitative synthesis was neither feasible nor appropriate.

For clinical tests to have genuine diagnostic utility, they must be reliable. A test can be considered reliable only if it is both valid and reproducible. Reliability refers to the consistency with which a measurement is performed [2]. A diagnostic procedure is therefore considered highly reliable when it produces consistent results under similar conditions. This implies that the same examiner at different times, or different examiners, should be able to correctly apply and interpret the same test. However, it must be emphasized that reproducibility alone does not guarantee test validity.

The reproducibility of a diagnostic test is defined as the degree of agreement among repeated assessments performed on the same subject. In the context of clinical palpation, intra-examiner agreement refers to repeated evaluations conducted by the same examiner at different times, whereas inter-examiner agreement involves evaluations performed by different examiners. Studies on palpation have shown that intra-examiner agreement is generally higher than inter-examiner agreement [3]. When assessing reliability, inter-examiner agreement is of greater relevance than intra-examiner agreement, as it better reflects the consistency of the procedure across different clinicians [4].

The validity of a diagnostic procedure refers to the extent to which it accurately measures what it is intended to assess. More precisely, validity is determined by comparing the performance of the procedure with a recognized gold standard or reference test. The focus on the PSIS and on the standing execution of the test reflects their central role in the traditional performance and interpretation of the SFT as commonly applied in clinical practice. Although specific imaging and clinical tests lack definitive diagnostic accuracy, image-guided intra-articular anesthetic injections are widely regarded as the most accurate method to confirm sacroiliac joint pain, often using predefined pain relief criteria as reference [5]. However, a test lacking sufficient reliability is ultimately ineffective, as it fails to provide consistent measurements of the variability it aims to investigate, such as symmetry of pelvic landmarks [6].

Given the central role historically attributed to PSIS palpation in osteopathic and manual therapy practice, Cooperstein and Hickey [3] conducted a systematic review of the literature addressing intra- and inter-examiner reliability in identifying PSIS position and assessing potential bilateral asymmetries between the right and left sides. Three studies evaluated PSIS palpation in the seated position [7-9], six in the prone position [10-15], three in the standing position [16-18], and one study included both seated and standing assessments [19]. The standing position was intentionally selected because it represents the functional context in which the SFT is most commonly applied, integrating gravitational load, lumbopelvic rhythm, and lower limb mechanics, thereby maximizing its clinical relevance. Ten studies [7-10,12-14,17-19] required examiners to bilaterally assess the PSIS, determining whether the structures were level within the same coronal plane or displaced relative to one another. Two studies [15,16] instead focused on localizing a single PSIS, assessing examiner agreement based on the distance between identified points, without applying specific statistical analysis methods. One additional study [11] measured inter-examiner agreement based on the median distance between bilaterally drawn lines over the PSIS.

From a reliability standpoint, the available evidence consistently indicates that none of the included studies achieved a level of inter-examiner reliability considered “substantial” according to the Landis and Koch scale [20]. Among the five studies that evaluated both intra- and inter-examiner reliability [7,10,11,14,15], higher reliability was consistently observed within examiners. In the eight studies that used kappa statistics to assess inter-examiner agreement, the weighted mean kappa value adjusted for sample size was 0.27 [3]. A trend toward a moderate correlation between kappa values and methodological study quality was observed, although it did not reach statistical significance (Pearson r = 0.43, p = 0.28), suggesting that higher-quality studies might demonstrate improved inter-examiner reliability [3]. Nevertheless, none of the studies reported a kappa coefficient equal to or greater than 0.60, the threshold indicating substantial agreement and potential clinical usefulness of the procedure according to Landis and Koch’s criteria [20]. Moreover, none reached even the minimum kappa level of ≥0.40, corresponding to moderate agreement. These findings are consistent with those of another systematic review [21] that evaluated intra- and inter-examiner reproducibility of other spinal palpatory landmarks.

According to the systematic review conducted by Seffinger et al. [22], neither examiners’ academic training, level of clinical experience, shared palpation methodology, nor the use of symptomatic subjects resulted in a significant improvement in the reliability of standard palpatory landmark assessment. Similar conclusions were reached by other authors [12,17], who reported that neither experience nor years of training improve the reliability of anatomical landmark palpation.

Accordingly, fundamental scientific doubts exist regarding the feasibility of using PSIS palpation to derive clinically meaningful information, beyond the well-documented poor reliability of the procedure. We share the concern raised by Liebenson and Lewit [23]: “The question about palpation’s reliability should not be turned against palpation, but should be turned towards asking how to develop reliable, responsive, and valid instruments?”

In this context, a recent critical review of the literature on pelvic palpatory tests [24] has highlighted the main conceptual and methodological limitations related to diagnostic interpretations of pelvic asymmetries, emphasizing the need to distinguish between the reliability of the palpatory act and the validity of the clinical construct being assessed, as well as the importance of orienting future research toward more functional interpretative models.

Throughout this review, historical osteopathic sources are explicitly discussed for their conceptual and educational relevance, whereas contemporary studies are referenced when addressing empirical evidence related to reliability, validity, biomechanics, or clinical utility

In light of these considerations, the present review aimed to determine, through an in-depth critical review, whether the SFT remains a test with potential clinical value and continued applicability in disciplines that use the hands for diagnostic and therapeutic purposes (manual therapy, osteopathy, chiropractic), or whether it should be abandoned altogether. This review will specifically address the following aspects: landmark acquisition technique; interpretation of results; training modalities aimed at improving examiner agreement; and considerations regarding the nature of the test itself.

## Review

Technique for PSIS assessment: what the literature suggests

**Figure 1:**
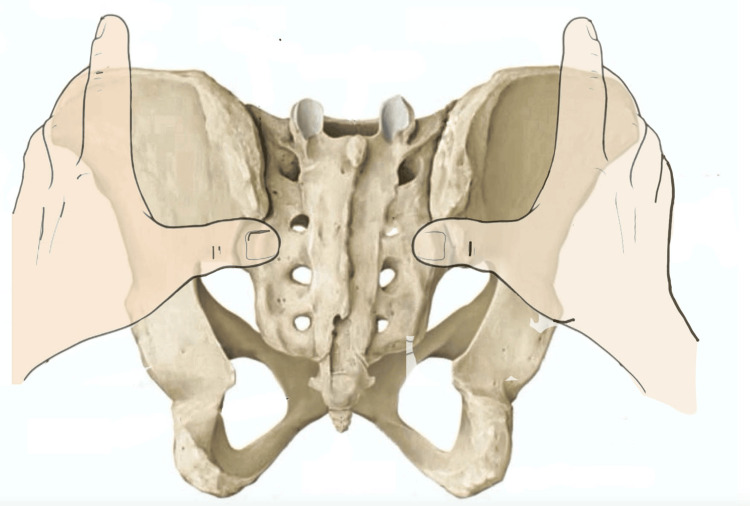
Position of the posterior superior iliac spine landmark acquisition during the standing forward flexion test. The image highlights the placement of the examiner’s thumbs with the fingertips positioned inferior to the posterior superior iliac spine. Image credit: Saverio Colonna.

The review by Cooperstein and Hickey (2016) highlights that, in clinical practice, examiners tend to palpate the PSIS primarily at its inferior aspect or at the point perceived as the most posterior [3]. However, the manner in which this palpation is performed remains largely described in anecdotal terms. The literature [19,25-27] suggests locating the PSIS at its inferior portion (Figure 1), yet no scientific evidence confirms that this represents the most accurate approach.

This assessment strategy appears to have a plausible anatomical rationale that warrants careful consideration. Specifically, the postero-inferior portion of the PSIS exhibits a more pronounced variation in bony contour, which may be more easily perceived by the examiner’s fingertips. However, this same region serves as the insertion site of the long dorsal ligament, which functionally connects the hamstring muscles to the PSIS and contributes to the control of anterior pelvic tilt.

When palpation is performed with the patient in the prone position, reduced ligamentous tension allows improved contact between the examiner’s fingers and the underlying bone. In contrast, during dynamic tests such as trunk flexion in standing, the tension generated in the long dorsal ligament may decrease direct bony contact by displacing the fingertips away from the PSIS surface.

Cooperstein and Hickey [3] also emphasize that, in some individuals, the PSIS may present a laminar morphology rather than a prominent bony projection, making the identification of a true “most posterior point” uncertain. For this reason, they propose an alternative approach: placing the index fingers on the lateral iliac crests while simultaneously sliding the thumbs into a region slightly superior and lateral to the PSIS. According to the authors, this method allows for more effective use of tactile sensitivity.

In a previous study [28], during examiner comparison conducted as part of training aimed at improving agreement, it was observed that the technique used for landmark acquisition itself could represent a confounding factor and may partly explain insufficient examiner concordance. In that work, training designed to improve agreement in PSIS palpation included palpatory sensitivity exercises, visual recognition of limb length discrepancy using laser devices, and practical application on anatomical models and human subjects, as previously suggested in another of our studies [24]. Additionally, as proposed by some authors [29], involving examiners in discussions regarding how to perform the data acquisition sequence to construct a procedure tailored to examiner needs revealed that reversing the order of assessment (reverse SFT), by starting data collection in trunk flexion and subsequently in standing, was more favorable.

Palpation of the PSIS, as proposed in the literature, targeting a point immediately inferior to the apex rather than the most posterior projection, may result, during trunk flexion, in separation of the fingertips from the underlying bony landmark due to tensioning of the long dorsal ligament. The hamstrings, particularly the biceps femoris [30], are in fascial continuity with the sacrotuberous ligament [30-33], which continues into the long dorsal ligament up to its insertion on the PSIS. During trunk flexion, increased hamstring tension, responsible for controlling anterior pelvic rotation [34-36], augments tension along this posterior myofascial chain, transferring forces toward the posterior sacroiliac complex. This mechanism may alter palpatory perception of the PSIS: during flexion, the examiner is more likely to lose contact with the landmark due to tissue tension variability and reduced pelvic stability [37]. Frequently, in an attempt to maintain contact with the bony landmarks, the examiner increases palpatory pressure, potentially compromising the subject’s balance. Such difficulties are generally less pronounced during the return to standing, a phase in which it is easier to maintain consistent palpatory pressure and more accurately localize the bony landmark [28].

**Figure 2:**
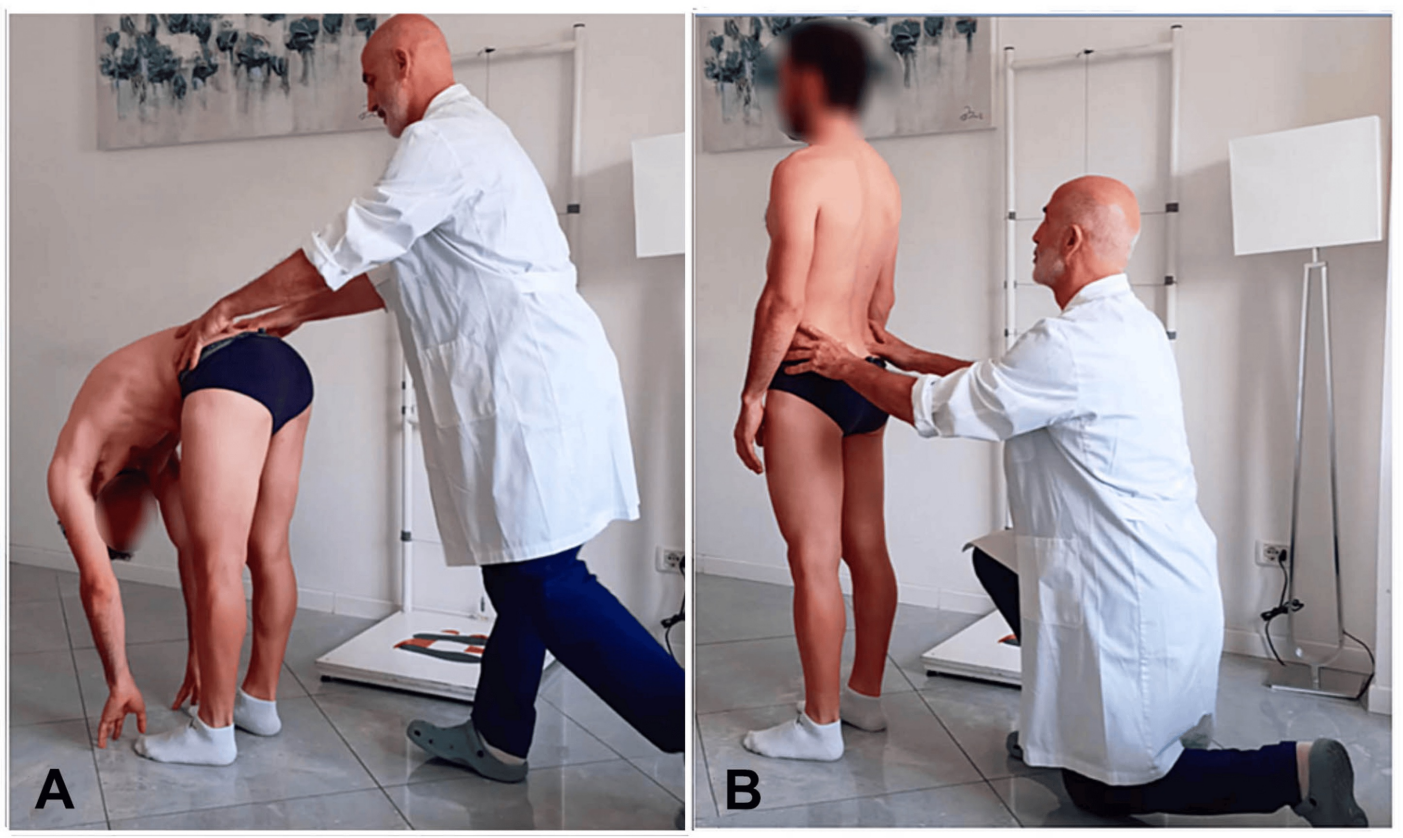
Reverse standing forward flexion test. (A) First posterior superior iliac spine assessment performed with the subject in trunk flexion. (B) Second posterior superior iliac spine assessment performed with the subject in the standing position. Image credit: Saverio Colonna. This figure was previously published in Cureus (Colonna and Mazzanti [24]) and is reproduced with permission.

The experience of the examiners in this study [28] indicated that initiating PSIS assessment with the subject in trunk flexion and subsequently in standing (Figure 2), that is, using a reverse SFT, resulted in greater accuracy in landmark acquisition and, consequently, improved agreement of findings. This phase aimed to enhance both intra- and inter-examiner reliability and establish stable methodological alignment before subsequent assessments.

The examiner records the relationship between the two PSIS during flexion and then during extension, comparing the two conditions. Interpretation of the test is addressed in the following section. Notably, the proposal to assess the PSIS during the trunk extension phase is not entirely new. Mitchell and Mitchell [38], in their book on muscle energy techniques, also suggested considering the initial phase of return from flexion, that is, extension, defining it as the new standing flexion test, in which the side that moves posteriorly first is considered positive.

Interpretation of results: what appears simple and linear in theory is not always so in practice

It is somewhat surprising that a test of this kind, one of the most frequently employed across different manual therapy disciplines for pelvic assessment, has a substantial body of literature addressing its reproducibility [7-10,12-14,17-19,27], yet virtually no studies specifically focused on the interpretation of its results. The widespread use of this test may reasonably be attributed to its ease of execution; however, the same cannot be said for result interpretation.

Traditional interpretations of SFT positivity assume that asymmetric cranial-ventral motion of the PSIS reflects hypomobility or dysfunction of the ipsilateral sacroiliac joint. According to this model, symmetrical PSIS motion is interpreted as a negative result, whereas greater cranial-ventral displacement of one PSIS during trunk flexion is considered indicative of sacroiliac dysfunction on that side [39]. However, this interpretative framework presupposes a direct and exclusive link between PSIS displacement and sacroiliac joint motion. However, available biomechanical evidence suggests that PSIS displacement during trunk flexion may result from multiple interacting factors, including hip mobility, lumbopelvic rhythm, myofascial stiffness, and pelvic morphology, rather than from isolated sacroiliac joint dysfunction alone. As a consequence, the simplified three-outcome model does not adequately capture the range of patterns observed in clinical practice.

**Figure 3:**
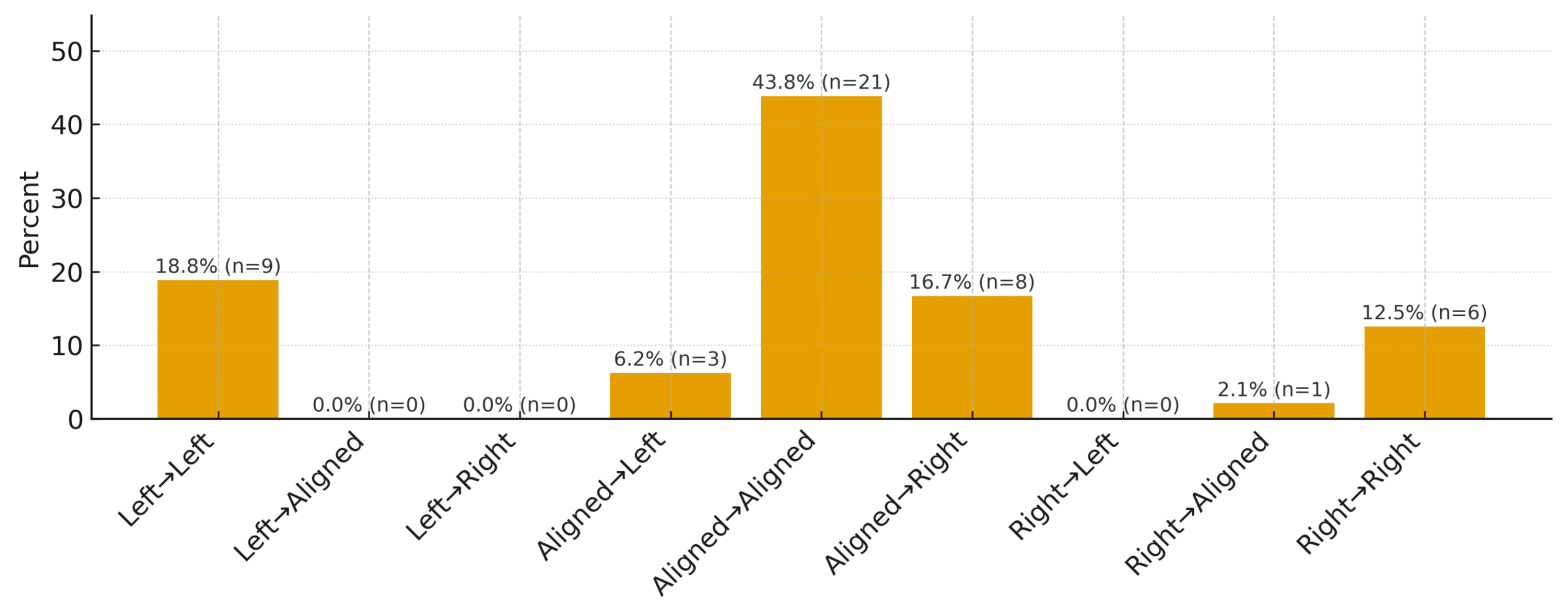
Posterior superior iliac spine assessment patterns during the standing forward flexion test. Analysis of posterior superior iliac spine (PSIS) assessments in 48 subjects evaluated by a single examiner. The first measurement represents PSIS assessment performed in trunk extension, whereas the second corresponds to assessment after trunk flexion. The first column (left–left), for example, indicates a left PSIS positioned more cranially in standing and remaining more cranial-ventral after trunk flexion; the second column (left-aligned) shows the left PSIS higher in standing with symmetrical PSIS alignment in flexion; the third column (left–right) indicates the left PSIS higher in standing and the right PSIS becoming more cranial-ventral in trunk flexion. Pattern nomenclature refers to PSIS position in standing followed by trunk flexion. “Aligned” indicates no detectable cranio-caudal asymmetry between PSIS. Image credit: Saverio Colonna.

In reality, the possible scenarios are nine rather than three. Figure 3 presents the outcomes derived from PSIS relative height measurements obtained in the standing position and compared with those recorded in trunk flexion, as observed in our previous study [28].

**Figure 4:**
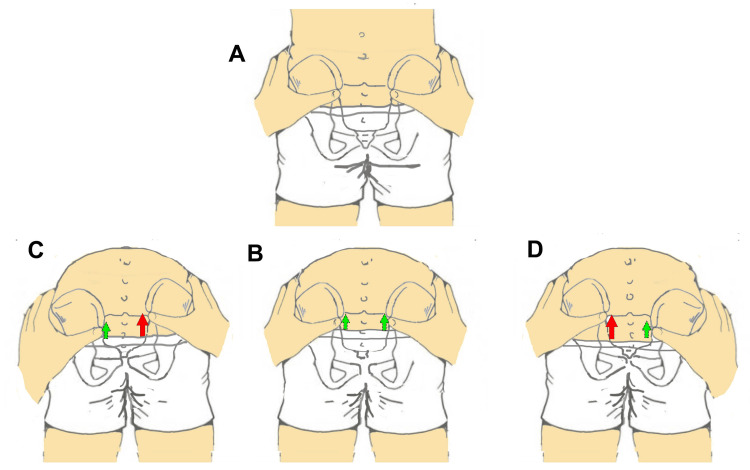
Posterior superior iliac spine patterns with clear clinical interpretation. When the posterior superior iliac spines (PSIS) are level in the standing position (A) and remain level during trunk flexion (B), the test result is interpreted as negative. When the PSIS are level in standing and become asymmetrical in flexion, with the right PSIS moving more cranial-ventral, the result is considered positive on the right; the opposite configuration corresponds to a positive result on the left (A and D). Image credit: Saverio Colonna.

The most straightforward and easily interpretable situations are three and are depicted in the central portion of the graph: aligned PSIS in standing and remaining aligned in flexion (central column of the graph); aligned PSIS in standing with greater cranial-ventral displacement to the right or left during trunk flexion (Figure 4). In the first case, the literature [27] considers the finding as negative for sacroiliac pathology; in the second case, when the PSIS start aligned in standing and greater cranial-ventral displacement of the right PSIS occurs during flexion, the test is interpreted as positive for right sacroiliac dysfunction; conversely, if greater cranial-ventral displacement is observed on the left side, the test is considered positive on the left.

**Figure 5:**
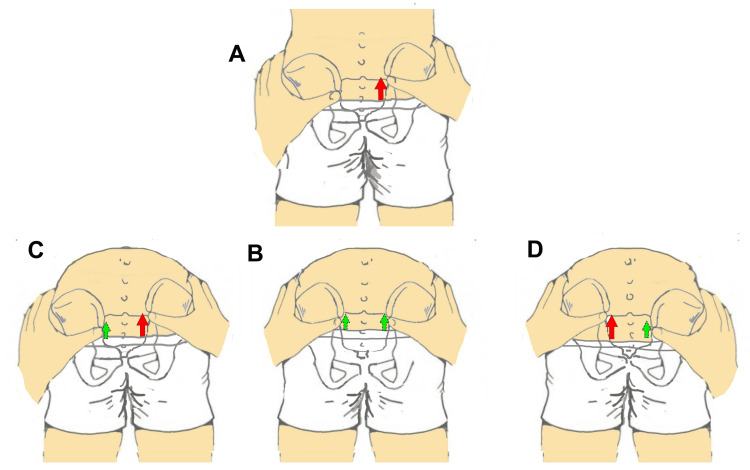
Posterior superior iliac spine patterns with ambiguous clinical interpretation. The right posterior superior iliac spine (PSIS) is positioned more cranial-ventrally in standing (A) and becomes level in trunk flexion (B). In another condition, the right PSIS is more cranial-ventral in standing (A) and remains more cranial-ventral during flexion (C). A clearly positive result on the left is observed when the PSIS configuration shown in standing in panel A is followed by the flexion pattern shown in panel D. Image credit: Saverio Colonna.

Situations outside these patterns are considerably more difficult to interpret. For example, how should a right-right pattern be interpreted (Figures 5A, 5C), in which the right PSIS is higher in standing and remains more cranial-ventral during trunk flexion? In this case, no PSIS has clearly undergone greater movement, as the right PSIS was already higher in the standing position. The same reasoning applies to a left-left pattern. Another scenario of uncertain interpretation is the right-aligned pattern: at the end of trunk flexion, the PSIS are aligned, but the left PSIS has traveled a substantially greater distance than the right (Figures 5A, 5B). A mirrored situation applies to a left-aligned pattern, in which the right PSIS moves more during flexion, yet both PSIS appear aligned at the end of the movement. Perhaps the least ambiguous scenario is the right-left pattern (Figures 5A, 5D), in which it is evident that the left PSIS has undergone a markedly greater cranial-ventral displacement.

As can be appreciated from practical application and not merely from theoretical considerations, situations arise, as frequently noted in the literature, that are difficult to interpret, at least from our perspective. An additional element supporting this viewpoint, partly previously highlighted in the literature [27], emerges from our prior work, where the highest level of agreement using the classical interpretation, defined as the PSIS that moves earlier and more during flexion relative to the contralateral side, reached a Kw of 0.82, whereas agreement for separate evaluation of the two phases reached Kw levels above 0.90. Thus, when PSIS symmetry in trunk extension and flexion is assessed separately, agreement is higher than when a global interpretation of the test is applied. This likely reflects the presence of ambiguous scenarios in the classical interpretation of the test, which leaves greater room for subjective interpretation.

Training modalities to improve examiner agreement

In recent studies reported in the literature, substantial emphasis has been placed on specific training protocols aimed at constructing examiner agreement when investigating the reproducibility of palpatory tests [10,24,29,40-43]. As previously discussed in an earlier study [24], in which doubts were raised regarding the modalities of training used to improve intra- and inter-examiner agreement, a fundamental conceptual error lies in considering these tests as purely “palpatory.” In reality, the hands only contribute to identifying the spatial position of the anatomical landmark, whereas visual input provides the spatial information required by the brain to generate the final diagnostic response. Providing a correct response with closed eyes would be impossible.

Based on these considerations, this type of assessment would be more accurately defined as a visuo-palpatory test rather than a purely palpatory one. However, if visual input contributes to, or even predominates in, the diagnostic process, how much time is actually devoted to visual training during preparation for palpation? Assessing whether an imaginary line connecting the hands or fingers placed on two anatomical landmarks, such as the PSIS, is horizontal or not is challenging and cannot be taken for granted.

Consequently, dedicating time to specific visual training is essential before conducting research on the reproducibility of visuo-palpatory tests [43], and even more so before training manual therapists. Training should also address the range of discrepancies that can still be considered symmetrical. Given that perfect alignment is rare, establishing how many millimeters of difference between two landmarks can still be regarded as a symmetrical posture or movement is crucial. Accurately identifying differences of 3, 4, or 5 mm is not straightforward without dedicated training using appropriate tools.

Furthermore, certain visual disorders should be excluded among participants, such as metamorphopsia, a condition first described by Foster and defined as a visual distortion in which a grid of straight lines appears wavy, and portions of the grid may appear missing [43]. An assessment of visual capabilities to exclude impairments in spatial plane recognition should therefore be recommended for anyone practicing manual therapy.

Conceptual confusion surrounding the SFT: from a functional tool to an inappropriate diagnostic test

For clinical tests to have genuine diagnostic utility, they must be reliable [44]. As previously stated, a test can be considered reliable only if it is both reproducible and valid. The validity of a diagnostic procedure represents the extent to which it effectively measures what it is intended to assess [5]. However, in the case of the SFT, what exactly is being evaluated?

Over time, the SFT has undergone a substantial conceptual drift from its original osteopathic foundations. The SFT was not designed to diagnose sacroiliac joint pathology but rather emerged as a palpatory assessment of functional asymmetry within the lumbopelvic complex. Over the decades, however, its application has progressively been reinterpreted, from a simple qualitative evaluation of pelvic coordination to a presumed medical diagnostic tool for sacroiliitis or sacroiliac pain, despite the absence of anatomical, biomechanical, or pathophysiological foundations to justify such use.

It has been demonstrated that the sacroiliac joint can represent a source of pain in individuals with low back pain; however, the etiology of this condition remains uncertain [45]. The relationship between sacroiliac joint dysfunction and sacroiliac pain is also unclear, as sacroiliac joint dysfunction is not necessarily symptomatic. Moreover, no valid biological mechanism has been identified to explain a mechanical dysfunction of the sacroiliac joint, and even the very existence of sacroiliac joint dysfunction remains debated, given that no clinical test has been shown to be both reliable and valid for its identification [46].

Content, construct, and criterion validity in palpatory tests: why the SFT cannot be interpreted as a diagnostic test

Recent literature on palpatory tests, including the systematic review by Najm et al. [47], has highlighted that the validity of a manual test can be articulated across three distinct dimensions: content validity, which refers to the theoretical adequacy of the test in relation to the phenomenon it intends to measure; construct validity, which evaluates whether the test behaves consistently with the underlying physiological or biomechanical models; and criterion validity, defined as the ability of the test to correlate with a recognized gold standard. Within this framework, it becomes evident why the SFT cannot be considered a diagnostic test for sacroiliac joint dysfunction or pathology.

From a content validity perspective, the SFT was historically conceived to identify functional asymmetries of the lumbopelvic complex rather than to detect joint pathology; its pathological interpretation is therefore conceptually inappropriate. Construct validity is also weak, as the kinematics of the sacroiliac joint do not exhibit an amplitude sufficient to justify palpably detectable differences in PSIS excursion, while numerous extra-articular factors (myofascial stiffness, pelvic morphology, and lumbopelvic rhythm) predominantly influence PSIS movement. Finally, criterion validity is absent; no study has demonstrated a significant correlation between the SFT and intra-articular anesthetic blockade or other objective measures of sacroiliac pain.

Consequently, the validity of the SFT is not medical-diagnostic in nature but rather functional: the test may describe movement patterns and biomechanical asymmetries, but it cannot identify sacroiliac pathology. This distinction is crucial to avoid interpretative errors and restore the SFT to its appropriate clinical use.

Osteopathic origins and functional rationale

In early osteopathic and manual medicine literature, authors such as Mitchell and Mitchell [38], Greenman [26], and Lee [48] described the SFT as a dynamic test aimed at identifying reduced mobility of one innominate relative to the sacrum. The test was considered positive when, during trunk flexion, the PSIS on one side moved cranially and ventrally earlier or to a greater extent than the contralateral side. This finding was interpreted as hypomobility or “restriction” of the ipsilateral innominate in nutation. Within this model, the SFT represented a functional assessment rather than an indicator of pathology.

Misinterpretation as a medical diagnostic test: origins of the confusion

Beginning in the 1990s, several clinical authors incorporated the SFT into test batteries for the diagnosis of sacroiliac pain, assuming that a palpatory asymmetry could correspond to joint pathology. The first study by Dreyfuss et al. [39], published in 1994, investigated the validity of the SFT in correctly identifying subjects with sacroiliitis by using healthy individuals to determine false negatives, thereby extending the test into the role of a medical diagnostic tool. Unsurprisingly, the authors concluded that asymmetry in sacroiliac motion due to relative hypomobility, as identified by these tests, may also occur in asymptomatic joints. Subsequent systematic reviews by Laslett [49,50] and Szadek et al. [51] further confirmed that motion or positional tests, including the SFT, demonstrate low reliability and no diagnostic value when compared with intra-articular anesthetic blockade, which is regarded as the gold standard for diagnosing sacroiliac pain.

From these studies emerged a consensus that only pain provocation tests, such as the thigh thrust, compression, distraction, and sacral thrust tests, show moderate reliability and acceptable sensitivity and specificity for identifying painful sacroiliac pathology, particularly when used in clusters [18]. However, such tests do not assess sacroiliac joint mobility, but rather its nociceptive response.

A conceptual comparison between the traditional diagnostic interpretation of the SFT and its functional reinterpretation is summarized in Table 1.

**Table 1:** Conceptual comparison between the traditional interpretation and the functional reinterpretation of the SFT. SFT = standing forward flexion test; SIJ = sacroiliac joint; PSIS = posterior superior iliac spine

Aspect	Traditional interpretation of the SFT	Functional reinterpretation of the SFT
Primary aim	Assessment of SIJ mobility	Assessment of lumbopelvic movement patterns
Type of outcome	Binary (positive/negative)	Graded or categorized asymmetry
Anatomical focus	Sacroiliac joint	Lumbopelvic system (SIJ, hip, spine, myofascial structures)
Biomechanical assumptions	Increased PSIS excursion reflects ipsilateral SIJ hypomobility	PSIS motion reflects integrated lumbopelvic and myofascial dynamics
Role of myofascial structures	Not considered or marginal	Central role in modulating pelvic motion
Influence of hip and spinal mobility	Generally neglected	Explicitly acknowledged
Diagnostic value	Presumed diagnostic test for SIJ dysfunction	Non-diagnostic, functional assessment tool
Clinical interpretation	Pathology-oriented	Movement- and function-oriented
Recommended use	Identification of SIJ dysfunction	Identification of functional asymmetries and movement strategies

The shift toward a fascial and lumbopelvic model

More recent biomechanical models, particularly those proposed by Snijders et al. [52], have shifted the focus from a sacroiliac joint considered inherently “unstable” toward a systemic, fascial interpretation of the lumbopelvic complex. The concepts of self-bracing and force closure have demonstrated that sacroiliac joint stability and its apparently abnormal motion largely depend on muscular and fascial tension rather than on true, measurable joint excursions. From this perspective, the asymmetrical PSIS movement observed during the SFT may reflect myofascial or postural dysfunction, such as involvement of the thoracolumbar fascia, hamstrings, or paraspinal muscles, rather than intra-articular dysfunction.

Moreover, according to the aforementioned authors [52], it is not reduced mobility that gives rise to symptomatic pathology, but rather excessive mobility. Consequently, the anecdotal logic of interpreting a positive SFT, based on greater PSIS motion, as evidence of ipsilateral sacroiliac joint restriction lacks a sound theoretical foundation.

The conceptual confusion primarily arises from an improper overlap between the concepts of dysfunction and pathology. In the original osteopathic model, the SFT was intended to identify functional movement asymmetries, that is, dysfunctions. Indeed, according to Mitchell and Mitchell [38], a positive SFT could indicate dysfunction of the sacroiliac joint or the pubic symphysis, without implying pathology or pain. It should be emphasized that, following the teachings of the founder of osteopathy, Dr. Andrew Taylor Still, osteopaths traditionally seek dysfunction rather than pathology [53].

In contrast, subsequent orthopedic literature [39,49-51] has interpreted the same test within a pathological paradigm, attempting to link SFT positivity to inflammatory or painful processes of the sacroiliac joint, an approach that has inevitably resulted in poor sensitivity, specificity, and diagnostic validity. Based on current evidence, the SFT may not be considered a diagnostic test for sacroiliac pain, but rather an observational tool useful for describing asymmetries of hip-pelvic-lumbosacral rhythm. Such asymmetries may arise from altered motion not only at the sacroiliac joint, but also at the hip joint, or may be influenced by myofascial factors.

For this reason, it would be appropriate to adopt, as proposed in the literature for other palpatory landmarks [40], a staged (categorized) assessment of asymmetry magnitude when present, rather than relying on the binary positive/negative response typically used in medical tests [54,55]. For the sake of completeness, although orthopedic tests are traditionally presented as dichotomous (positive/negative), some examinations used to assess anterior cruciate ligament instability adopt graded scales. The Lachman test is frequently reported as 1+, 2+, or 3+ according to anterior translation and endpoint quality, while the pivot-shift test is classified according to the International Knee Documentation Committee scale (0-3) [56]. This confirms the existence of ordinal paradigms even within orthopedic testing, despite their less frequent use compared with binary models.

Reconsidering the meaning of positivity in the SFT: biomechanical determinants beyond sacroiliac dysfunction

Traditional osteopathic interpretation models of the SFT associate the side exhibiting greater cranial-ventral movement of the PSIS with hypomobility or dysfunction of the ipsilateral sacroiliac joint [57,58]. However, multiple intrinsic and extrinsic biomechanical factors may influence PSIS motion during forward trunk flexion, calling into question the assumption that the side demonstrating greater movement necessarily reflects a “pathological” sacroiliac joint.

Hypothetical causes of PSIS asymmetry

**Figure 6:**
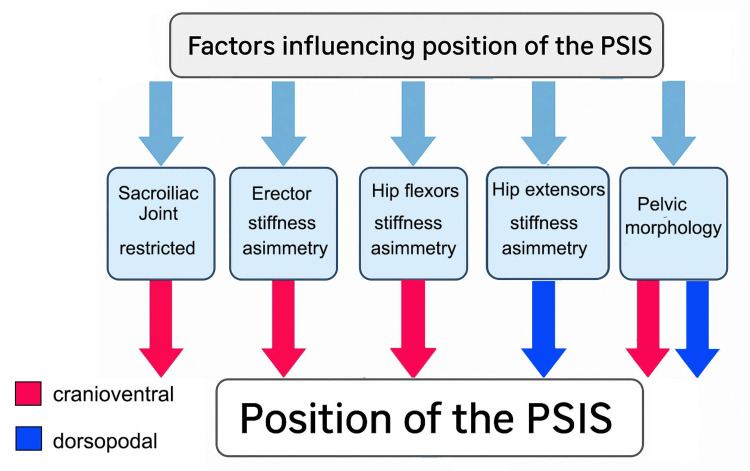
Factors that may influence PSIS position with their relative direction. Posterior superior iliac spine (PSIS) position may be influenced by articular, myofascial, and morphological factors. It cannot be excluded that more than one of these factors may act simultaneously. Image credit: Saverio Colonna.

The theoretical factors that may influence asymmetry in PSIS level are summarized in Figure 6 and include sacroiliac joint restriction; myofascial stiffness of the erector spinae; myofascial stiffness of the hip flexor muscles; myofascial stiffness of the hip extensor muscles; and morphological alterations of the pelvis. A multifactorial contribution to PSIS asymmetry may represent one of several possible explanatory models, rather than a single, specific causal mechanism.

Lower limb length discrepancy was intentionally excluded, as the literature does not support the existence of a single, invariant pattern of pelvic compensation in the presence of leg length discrepancy. Rather, most studies converge in describing a three-dimensional pelvic adaptation commonly defined as torsion [59,60], in which frontal-plane obliquity is associated with relative adjustments of the iliac bones in the sagittal and transverse planes [61,62]. A tendency toward anterior rotation of the innominate on the side of the shorter limb and posterior rotation on the side of the longer limb has been reported with some frequency [63-65], particularly in cases of mild-to-moderate discrepancy; however, this pattern is not universal and is strongly dependent on the measurement method employed [66].

Nevertheless, the lowering of the PSIS and, more generally, of the iliac bone due to a shorter limb could be limited by the anterior rotation associated with physiological compensatory mechanisms. This may help explain why, in the study by Sutton et al. [17], the authors concluded that inter-examiner reliability in detecting artificially induced PSIS asymmetries, produced by inserting a 5 mm heel lift under one foot, was poor. Their final recommendation was to reconsider the inclusion of this osteopathic model within osteopathic training programs.

Influence of hip joint mobility

During optimal trunk flexion, the hip joint contributes approximately 65% of the overall movement [67,68]. Limitations of the hip joint, for example, due to osteoarthritis, may alter the normal coupling between the femur and the pelvis during trunk flexion. The pelvis on the side of the stiffer hip tends to undergo less anterior rotation, while the contralateral iliac bone compensates with greater anterior rotation, generating an apparent positive SFT on the more mobile side, according to the classical interpretation previously described. This mechanical compensation may therefore reflect an asymmetry in hip motion rather than sacroiliac joint dysfunction.

Myofascial restrictions

**Figure 7:**
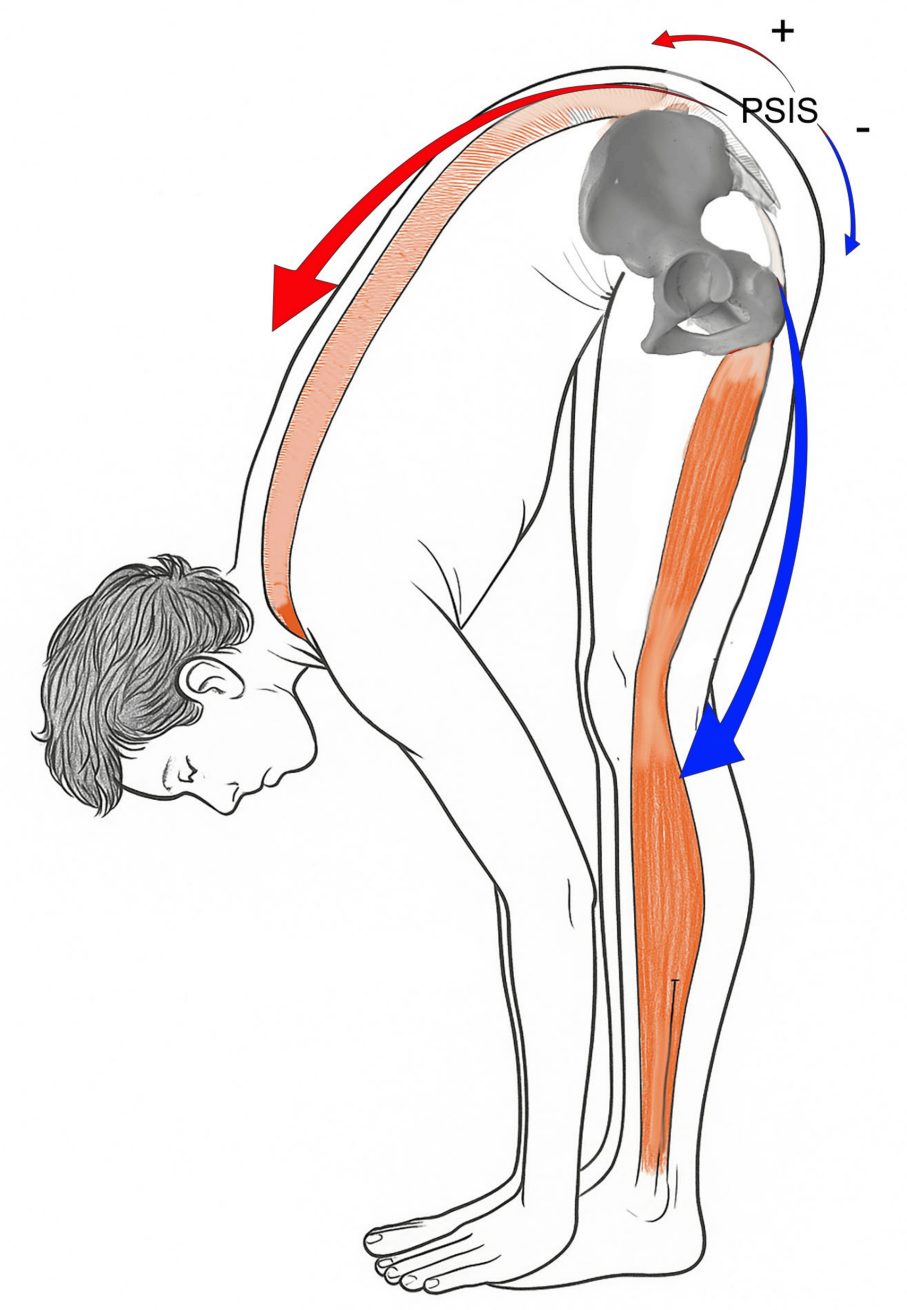
Schematic representation of fascial dysfunctions potentially influencing posterior superior iliac spine position. The red arrow indicates the action of cranial myofascial systems (paraspinal structures), whereas the blue arrow represents the influence of caudal myofascial components (hamstrings). Image credit: Saverio Colonna.

With regard to myofascial influences on the SFT, two dysfunctional hypotheses may be considered: caudal factors and cranial factors (Figure 7).

Influence of Hamstring Tightness

Clinical observations have suggested that stiffness of the posterior thigh muscles influences lumbopelvic rhythm [69]. Restrictions in hip motion or postural asymmetries at the hip level lead to compensatory movement patterns of the lumbar spine and, consequently, to increased stress on spinal soft tissues and a higher risk of low back pain [35].

The possibility that the SFT may be influenced by asymmetric hamstring stiffness, affecting the anterior advancement of the iliac bone contralateral to the PSIS that moves more cranio-ventrally, has been reported in the literature for a long time [37]. The functional role of the hamstrings during this specific task, i.e., forward trunk flexion in standing, may be integrated into the broader fascial systems of the lower limb, extending as far as the plantar fascia [70,71]. This type of influence can be classified among caudal dysfunctions.

Influence of Paraspinal Muscle Retraction/Stiffness

While eccentric contraction of the hip extensors, among which the hamstrings play a major role in controlling pelvic inclination, eccentric contraction of the spinal erector muscles at the beginning of trunk flexion controls spinal flexion. At a specific point during trunk flexion, these muscles relax as part of the flexion-relaxation phenomenon (FRP) [72]. Numerous studies have demonstrated that the FRP represents a consistent and predictable response in most healthy individuals without low back pain. Reduced flexibility of the soft tissues surrounding the lumbar spine results in an abnormal lumbopelvic rhythm and loss of the FRP [67,73].

However, if during trunk flexion a portion of the paraspinal muscles maintains activation [74,75], it is reasonable to assume that, on that side, greater cranial traction may occur at the sites of attachment of the erector spinae on the sacrum and ilium [76], potentially manifesting as a more cranial-ventral position of the PSIS. These findings support the rationale that side-to-side differences in paraspinal stiffness or activation may modulate an asymmetric right-left lumbopelvic rhythm and, clinically, contribute to a positive SFT ipsilateral to the side of increased stiffness. This type of influence can be classified among cranial dysfunctions.

Influence of Hip Flexor Retraction: Iliacus and Rectus Femoris

MRI studies have shown that the primary hip flexor muscles frequently exhibit asymmetric morphology between the right and left sides. Sanchis-Moysi et al. [77] documented that, in sedentary individuals, iliopsoas volume is on average approximately 4% greater on the dominant side, whereas in professional tennis players, this asymmetry is reversed, with a 13% hypertrophy on the non-dominant side [77].

Subsequent analyses of hip muscle cross-sectional area have confirmed the presence of lateral differences in iliopsoas volume across different populations, including large cohorts from the UK Biobank [78] and elite athletes [79,80]. Overall, these data indicate that a certain degree of structural asymmetry of the hip flexors is common even in asymptomatic individuals and may contribute, together with differences in stiffness and neuromuscular control, to generating asymmetric tilt of the hemipelvis and, consequently, PSIS misalignment in the standing position.

Although no study has yet directly measured unilateral hip flexor stiffness in relation to PSIS asymmetry, these findings support the hypothesis that an imbalance between anterior and posterior pelvic rotators across the two hemipelves may generate different pelvic torsions, thereby contributing to PSIS asymmetry in standing.

Structural morphology of the pelvis

In addition, the pelvis itself exhibits structural morphological asymmetries. Findings from a study [81] reported that 157 out of 159 pelvic rings (98.74%) assessed using CT showed a maximum side-to-side deviation greater than 4 mm, and 27 cases (16.98%) exceeded 10 mm. Some studies have concluded that the pelvis is not completely symmetrical in any individual; asymmetry represents the norm rather than the exception [82]. These findings suggest that variations in pelvic morphology may significantly influence measurements of pelvic tilt and anonymous rotational asymmetry [83].

Implications for clinical interpretation

Using the SFT exclusively to assess greater or lesser sacroiliac joint mobility entails inherent mechanical limitations. The actual range of motion of the sacroiliac joint is extremely limited: radiostereometric studies report angular motions in the order of 0.2°-0.6° and translations of only a few millimeters [84]. Radiostereometric investigations have consistently demonstrated that SIJ mobility is minimal, with mean rotations of approximately 2.5° (0.8-3.9°) and translations as small as 0.7 mm (0.1-1.6 mm), even under load or in extreme positions [84].

In contrast, clinical and anatomical investigations show that pelvic and PSIS asymmetries commonly reach values of several millimeters. Levangie used a 4-mm threshold to classify PSIS height differences in standing and sitting during pelvic torsion tests, noting that many individuals exceeded this limit and that the association with sacroiliac dysfunction was weak [58]. More recently, Yu et al. quantified asymmetry in the PSIS-to-floor distance as a relevant parameter of pelvic asymmetry in individuals with chronic nonspecific low back pain [85], while three-dimensional analyses of the pelvic ring have revealed morphological asymmetries ranging from 4 to 10 mm at multiple anatomical sites, including the PSIS, in the vast majority of samples [81].

Overall, these data render it biomechanically implausible that centimeter-scale differences in PSIS height observed during the SFT directly reflect the sub-millimetric mobility of the sacroiliac joint. It is more likely that the test captures a combination of pelvic morphology, global pelvic tilt, soft tissue deformation, and measurement error, rather than isolated sacroiliac joint mobility [27].

Considering these biomechanical interdependencies, the SFT should not be interpreted solely as a direct measure of sacroiliac mobility. The side identified as “positive” may result from compensatory movements involving the hip, hamstrings, or paraspinal muscles, each contributing differently to lumbopelvic rhythm. Therefore, SFT positivity should be regarded as an indicator of asymmetry in lumbopelvic kinematics rather than as a direct diagnosis of sacroiliac dysfunction, and even less so of sacroiliac pathology.

Reduced unilateral PSIS mobility during trunk flexion in the SFT may therefore indicate stiffness of the myofascial systems responsible for hip and spinal extension, or, more appropriately, an imbalance in tension between posterior and anterior pelvic rotator systems. These systems include muscles such as the hamstrings, gluteus maximus, paraspinal muscles (erector spinae and multifidus), iliacus, and rectus femoris.

As reported in the literature [86], hamstring extensibility, quantified using the passive straight leg raise, significantly influences pelvic inclination and spinal flexion during maximal forward trunk flexion with extended knees, but not in standing or cycling positions, highlighting a functional relationship between posterior thigh muscle stiffness and pelvic retroversion.

While hamstring extensibility appears to influence pelvic position and PSIS inclination during trunk flexion, standing posture, where active elongation of the posterior chain does not predominate, may be primarily modulated by the tone and stiffness of the gluteus maximus. Alvim et al. [87] described the importance of this muscle in controlling hip extension and stabilizing the pelvis. In this context, asymmetry in gluteal stiffness between sides may generate differential extensor leverage and, consequently, different orientations of the two hemipelves, reflected in PSIS asymmetry in standing.

Several studies also suggest that stiffness or shortening of the hip flexors may influence pelvic inclination in standing. For example, Preece et al. [88] demonstrated that stretching of the hip flexors reduces anterior pelvic tilt in standing, indicating an inverse relationship between flexor length and pelvic tilt. Ludwig et al. [89] further showed that targeted muscular strategies addressing the hip flexors can modify pelvic posture. Kim and Shin [90] reported that increasing iliopsoas tone is associated with increased anterior pelvic tilt, whereas posterior pelvic tilt is associated with increased hamstring tension. Klee et al. [91] demonstrated that individuals with well-developed abdominal muscles exhibit reduced pelvic anteversion, while those with strongly developed hip flexors display increased anterior pelvic tilt.

Similarly, increased passive stiffness of the rectus femoris has been proposed to promote anterior pelvic tilt during movement and excessive lumbar extension [92]. The same applies to the multifidus and erector spinae muscles, which have been identified as contributors to increased pelvic inclination [90,93].

A substantial body of evidence indicates that the primary myofascial systems responsible for lumbopelvic control do not exhibit reliable bilateral symmetry. Hamstrings frequently show significant side-to-side differences in length, stiffness, and strength between dominant and non-dominant limbs, both in healthy individuals and in athletes involved in asymmetric sports [94,95]. Similarly, the gluteus maximus may present marked asymmetries in strength and activation, with direct effects on pelvic orientation [87,96]. Hip flexors also demonstrate unilateral variations in thickness, tone, or stiffness, associated with lateral differences in pelvic tilt and hip kinematics [77,78,97].

Taken together, these findings support the hypothesis that relative PSIS height in standing or during trunk flexion largely reflects the balance of myofascial tensions, rather than intrinsic sacroiliac joint mobility, and that asymmetries in these systems may produce differential inclinations of the two hemipelves.

Differential diagnosis between myofascial retraction dysfunctions

**Figure 8:**
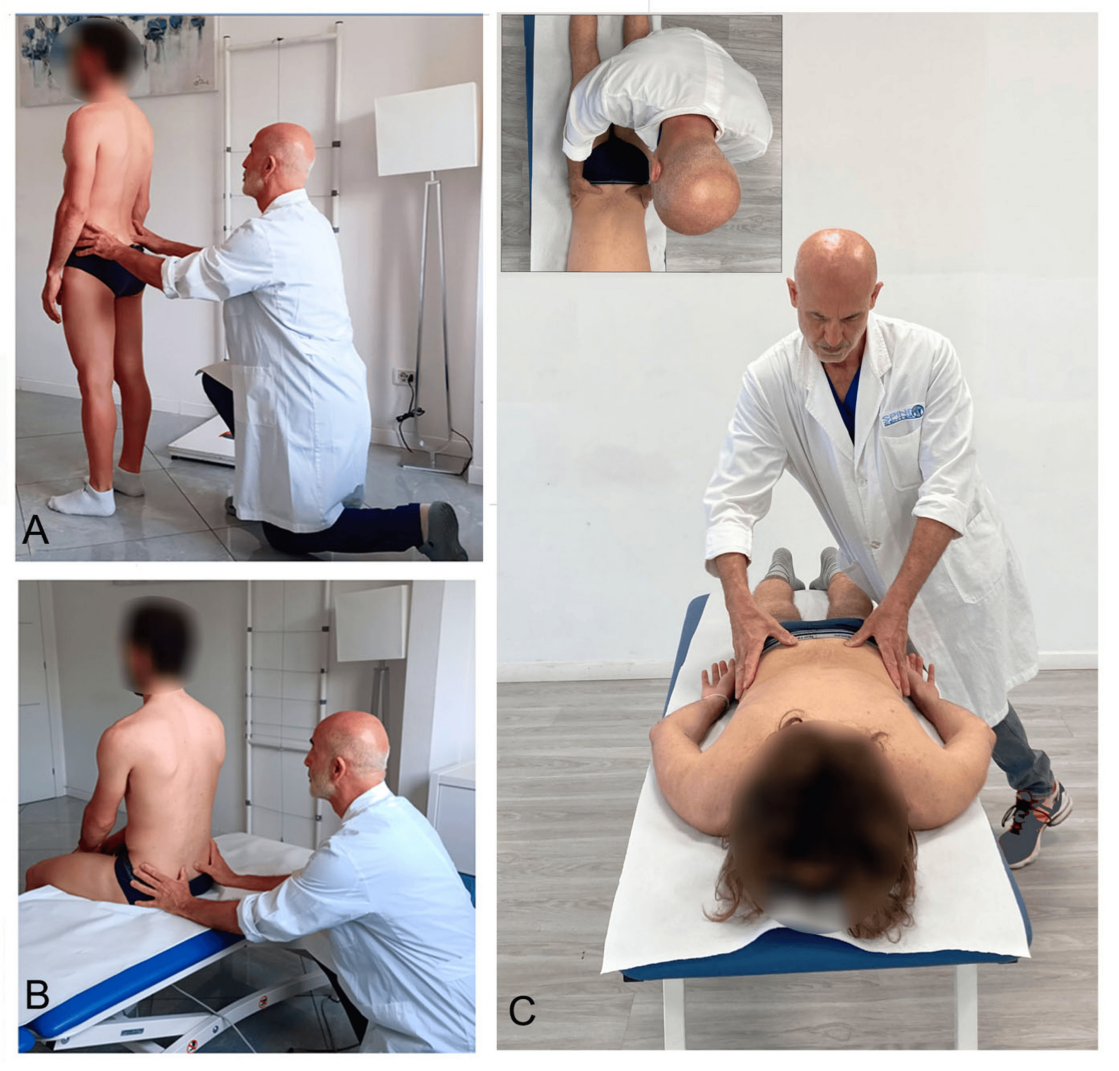
Example of palpatory assessment of the posterior superior iliac spine in subjects evaluated in different postures. (A) Standing position; (B) seated position; (C) prone position. The inset shows a superior view of posterior superior iliac spine palpation performed in the prone position. Image credit: Saverio Colonna. Part of this figure (A) was previously published in Cureus (Colonna and Mazzanti [24]) and is reproduced with permission.

An asymmetry in PSIS position may be either structural (pelvic morphology) or functional (myofascial asymmetry). To help identify which of these factors predominates, and whether a functional asymmetry arises from structures caudal or cranial to the PSIS, it may be useful to combine the SFT (Figure 8A) with the seated flexion test [26,27,38] (Figure 8B).

Compared with the standing position, trunk flexion mechanics in sitting change substantially: the load transfer point shifts from the plantar surface of the feet to the ischial tuberosities and posterior thighs. In this position, the hamstrings, due to knee flexion, operate under mechanical disadvantage relative to the gluteus maximus, which remains a key muscle for controlling pelvic anteversion against gravity.

If, during the seated flexion test, the PSIS relationship remains unchanged compared with the standing SFT, it may be logically inferred that the asymmetry is more likely due to pelvic morphological asymmetry or to a cranial myofascial dysfunction (i.e., involving paraspinal systems). Conversely, if PSIS asymmetry is reduced or abolished in sitting, the altered iliac movement may be primarily attributed to caudal myofascial systems, in which the hamstrings play a major role.

For indirect assessment of myofascial dysfunctions via PSIS asymmetry, the SFT should not be used dichotomously (positive/negative, right/left), but rather in a categorized, multi-level manner, as previously proposed [28]. Only this approach allows clinically meaningful comparisons across the three assessment conditions of PSIS levels (standing, sitting, and prone), enabling better classification of the potential origins of myofascial dysfunction.

In cases where the seated flexion test remains positive, differentiation between hypothesized pelvic morphological asymmetry and asymmetric cranial fascial traction from the paraspinal erector system may be further explored using PSIS palpation in the prone position (Figure 7C). In this position, paraspinal traction is expected to be reduced, whereas pelvic morphological asymmetry would remain unchanged. Thus, if PSIS asymmetry persists consistently across standing, sitting, and prone assessments, it may reasonably be attributed to pelvic morphology. Conversely, if PSIS asymmetry is substantially reduced or abolished in prone positioning, a cranial myofascial dysfunction may be hypothesized.

Because the PSIS position in static posture and during trunk flexion primarily reflects the balance of myofascial tensions of the hip and pelvis, which are physiologically asymmetric in most individuals, the interpretation of the SFT as a direct measure of SIJ mobility is methodologically unsustainable.

For clinical application of these tests, potential interference from the hip joint should not be overlooked, particularly reduced mobility due to degenerative changes. Indeed, several kinematic studies have documented that hip osteoarthritis is associated with reduced pelvic excursion during functional activities. Patients with hip osteoarthritis show a limited range of motion and a “stiff” coordination pattern between pelvis, femur, and hip, especially during deep flexion or rotational postures [98,99]. In prosthetic contexts, hip stiffness has likewise been shown to reduce pelvic mobility, whereas surgical intervention tends to partially restore pelvic excursion [100]. Accordingly, before using the SFT for myofascial assessment, mechanical alterations of the hip joint (e.g., osteoarthritis, femoroacetabular impingement) should be excluded using appropriate clinical tests.

## Conclusions

The SFT remains widely used in manual therapy and osteopathic practice, despite long-standing concerns regarding its clinical interpretation. This critical narrative review suggests that many of the limitations attributed to the SFT are less related to the palpatory procedure itself than to the diagnostic meaning traditionally assigned to its findings. When applied without standardized procedures or adequate training, the test demonstrates poor inter-examiner reliability; however, available evidence indicates that reproducibility may improve when structured and specific training protocols are implemented. From a biomechanical and anatomical perspective, the minimal intrinsic mobility of the sacroiliac joint, the high prevalence of pelvic morphological asymmetries, and the influence of hip mobility, as well as cranial and caudal myofascial tensions, challenge the assumption that SFT findings can be directly interpreted as indicators of sacroiliac joint pathology. Within this context, the SFT shows important limitations with respect to content, construct, and criterion validity when used as a diagnostic test for sacroiliac joint dysfunction. Conversely, when reframed within a functional and non-diagnostic model, the SFT may provide clinically relevant information regarding asymmetries in lumbopelvic kinematics and movement strategies during trunk flexion and extension. Rather than identifying sacroiliac joint dysfunction per se, the test appears to reflect the integrated behavior of the lumbopelvic complex, influenced by joint, muscular, fascial, and morphological factors. These considerations support moving away from a dichotomous positive/negative interpretation of the SFT toward a graded and context-dependent assessment. Interpreted in this manner, the SFT may retain value as a functional observational tool within a comprehensive biomechanical evaluation, while its use as a stand-alone diagnostic test, or as a confirmatory tool in association with provocation tests, for sacroiliac pathology, should be approached with caution. Future research should aim to correlate SFT findings with three-dimensional kinematic analysis, myofascial stiffness measures, and standardized training protocols to better clarify its functional significance.
